# Psychometric Properties of the SEQ-W Scale: An Instrument for the Estimation of Sexual Harassment in the Workplace

**DOI:** 10.3390/ejihpe15060101

**Published:** 2025-06-05

**Authors:** M. Isabel Soler-Sánchez, José Antonio López-Pina, Mariano Meseguer-de Pedro

**Affiliations:** 1 Department of Psychiatry and Social Psychology, Faculty of Psychology, University of Murcia, 30100 Murcia, Spain; misoler@um.es; 2 Department of Basic Psychology and Methodology, Faculty of Psychology, University of Murcia, 30100 Murcia, Spain; jlpina@um.es

**Keywords:** sexual harassment at work, SEQ-W, validation, exploratory factor analysis

## Abstract

(1) Background: Sexual harassment in the workplace is a problem that particularly affects women and is not an exception in the world of work. Factors such as power asymmetry, the predominantly masculinized culture in many organizations, and the potential impunity of perpetrators increase the associated risks. This study aims to analyze the psychometric properties of the Sexual Experiences Questionnaire-Workplace (SEQ-W) to measure sexual harassment at work and assess its validity in Spanish. (2) Methods: A total of 120 active female workers (67.5% European and 32.5% South American) participated, completing validated instruments to measure sexual harassment, workplace bullying, self-perceived health, and job satisfaction. The questionnaires were administered online, ensuring anonymity and explaining the study’s objectives. (3) Results: An exploratory factor analysis revealed a bifactorial structure with the dimensions “Sexual Harassment by Coercion” and “Harassment by Unwanted Sexual Attention.” Both dimensions demonstrated adequate internal consistency, concurrent validity with workplace bullying and well-being scales, and discriminant validity with job satisfaction. Additionally, a pronounced floor effect was observed, indicating a low prevalence of sexual harassment in the sample. (4) Conclusions: the SEQ-W scale is a useful tool for assessing sexual harassment among active Spanish-speaking female workers, considering its validated bidimensional structure in Spanish.

## 1. Introduction

Sexual harassment at work is a matter of concern in practically all countries (da Silva Fonseca et al., 2018). This problem began to be studied in the 1960s in the USA, when feminist movements highlighted the need to make visible and denounce the behaviors suffered by women entering the workplace (Baker, 2007). Since then, it has been approached from both a normative and a psychological perspective.

From a legal perspective, there are various international regulations aimed at preventing, intervening and punishing this type of violence. These include the Declaration of Philadelphia of the International Labor Organization (ILO), the United Nations Convention on the Elimination of All Forms of Discrimination against Women, and the Sustainable Development Goals (SDGs) of the same organization, particularly SDG 5, which promotes gender equality, and SDG 8, which calls for fair and safe working conditions (Women UN, 2019, 2020). At the national level, each country has incorporated specific regulations into its labor legislation to address this issue. In Spain, (Organic Law 10/2022) current legislation on comprehensive protection of sexual freedom defines sexual harassment as conduct that creates an objectively humiliating, hostile or intimidating situation for the victim through expressions, behavior or propositions of a sexual nature that do not constitute other more serious offenses.

From the psychological perspective, Fitzgerald et al. (1997) defined sexual harassment at work as “unwanted sexual-type behaviors at work that are perceived by the person on the receiving end as offensive, exceeding his or her coping resources, or threatening his or her well-being” (p. 15).

It is widely accepted that sexual harassment originates mainly from organizational conditions that facilitate its occurrence (Fitzgerald et al., 1995; Karami et al., 2021). Antecedents of sexual harassment in the workplace include job temporality, the physical nature of the job, negative relationships with co-workers and/or supervisors, as well as aspects of organizational culture and gender composition (Riddle & Heaton, 2023).

Sexual harassment has a very significant impact on those who are harassed (Women UN, 2019). The consequences of this harassment include feelings of irritation, anger, fear, humiliation and stress (McCann, 2005) and can lead to psychological illnesses, depression being one of the most common; thus Diez-Canseco et al. (2022) in a meta-analysis revealed, a prevalence of depression of 26%, with a significant increase in the risk of depression (2.69 higher), influenced by factors such as the frequency of harassment and the involvement of coworkers. Furthermore, in another meta-analysis, Willness et al. (2007) related sexual harassment experiences with decreased job satisfaction, organizational commitment, job abandonment, poor physical and psychological health, and even with the presence of Post-Traumatic Stress Disorder (PTSD) symptoms.

Fitzgerald et al. (1988) developed a theoretical model of sexual harassment on which they based the subsequent construction of the SEQ scale. This model stems from both legal and psychological approaches and is composed of three related but conceptually distinct dimensions: sexual coercion, unwanted sexual attention, and gender harassment. The first dimension, sexual coercion, constitutes the clearest example of what has traditionally been understood as sexual harassment, extortion or sexual cooperation in exchange for favors in the work context, such as promotions, for example. This dimension would be related to the quid pro quo perspective. The second, unwanted sexual attention, includes a wide variety of offensive and non-reciprocal behaviors, both verbal and non-verbal. Finally, the third dimension, gender-based harassment, refers to a series of behaviors that are not always aimed at maintaining explicit sexual relations, but to a series of forms of sexual exhibitionism as displays of gender superiority (it would include insulting and degrading behaviors, hostile attitudes towards women, distribution or display of pornographic material, etc.). This seems to be the most common form of harassment in organizations; however, in most research it has been ignored and has focused mostly on the other two dimensions (Fitzgerald et al., 1995).

With this model in mind, Fitzgerald et al. (1988) published the first version of the 30-item Sexual Experiences Questionnaire based on the experiences of students in higher education, with three response options (never, once, and more than once). A sample of 468 students was used and after a psychometric analysis, two items were eliminated, so that a 28-item version was presented. Reliability was 0.86 (test–retest). This article also includes a second version (SEQ2) where 5 more items were added to the 30 items and administered exclusively to women, with a sample of 642 female university workers (professors or administration and management) where they achieved a reliability of 0.86.

Subsequently, the SEQ-W scale (Fitzgerald et al., 1995) was constructed, a 20-item version, with a five-point Lickert-type response system where it is administered to a sample of 1156 workers of a state service company after a factor analysis is reduced to 17 items and presents three dimensions: harassment based on sex (α = 0.82), unwanted sexual attention (α = 0.85) and sexual coercion (α = 0.42).

In addition to the various versions of the SEQ (Gutek et al., 2004), there is currently a short version (SEQ-s) containing eight items. There is also a revised version designed for use by women and men in the Armed Forces (SEQ-DoD) that consists of 16 items in its final version (Fitzgerald et al., 1999). In the Spanish-speaking population (SEQ-L) a validation was carried out for Mexican-American women (Cortina, 2001) with 25 items and a factor structure of three dimensions. In South America, research such as that by Merkin (2012) shows that the perception and reporting of sexual harassment are strongly influenced by social norms about power and gender, which can modify the expected factorial structure. In Spain and other European countries, the visibility and reporting of harassment are more institutionalized, although cultural differences persist with respect to South America in terms of perceptions of gender (Lombardo & Bustelo, 2022). These sociocultural differences can influence both the reported prevalence and the interpretation of the items, justifying the need for cultural and linguistic adaptations of the instrument.

Nevertheless, the SEQ-W questionnaire can be a valuable tool in the assessment of sexual harassment in women workers. To ensure its applicability and accuracy in Spanish-speaking contexts, it needs to be validated in Spanish. Cultural and linguistic adaptation is crucial for the questionnaire to accurately capture the experiences of women in specific work settings and to reflect cultural subtleties that may affect the perception and expression of sexual harassment.

The main objective of this paper is to analyze the psychometric properties of the SEQ-W (Fitzgerald et al., 1995) scale aimed at estimating sexual harassment at work, in a sample of Spanish-speaking working women.

## 2. Method

### 2.1. Participants

A convenience sample of 120 active female workers with a mean age of 34 years (SD = 10.60). The 67.5% are European, while 32.5% are of South American origin. Some 33.6% are single, 33.5% are married and 17.6% are cohabiting. Most of the sample had a university education (72.5%), followed by those with secondary education (22.5%) and primary education or no education (5%). The majority have a permanent employment contract (58.3%), and 37.5% of the participants have a temporary contract. The average length of service in the company is 6 years (SD = 6.45), while in the job, it is 4 years (SD = 4.37). The socio-demographic characteristics of the group are shown in Table 1.

### 2.2. Procedure

A member of the research team oversaw in charge of delivering the questionnaires to active female workers. The questionnaires contained sociodemographic questions and self-report scales related to the study variables. They were informed of the objectives of the study and that the study was anonymous and voluntary. Likewise, a statement of informed consent was given in which the participants expressed their willingness to participate in the study. A total of 155 questionnaires were distributed and 124 were returned, of which 4 were rejected because they were not correctly completed (response rate 77.4%).

### 2.3. Instruments

Sexual harassment at work was estimated with the SEQ-W scale of Fitzgerald et al. (1995). The scale was originally in English, so firstly, a back-translation of the questionnaire was performed. First, it was translated from English to Spanish, and then a native speaker was asked to translate it from Spanish to English to verify the level of coincidence of the translation. This instrument is composed of 17 items related to behaviors related to the presence of sexual harassment at work. The response scale is Likert-type with 5 response options (1: Never, 5: Always). An example item is “During the last two years, how often did a supervisor or co-worker make ordinary sexual comments to you” (item 2).

For the measurement of workplace bullying, the Einarsen and Raknes (1997) NAQ bullying scale was used in the Spanish adaptation of Soler-Sánchez et al. (2010). This questionnaire assesses perceived workplace bullying. To do so, it asks participants to answer how often in the last 6 months they have perceived each of the 24 hostile behaviors or “negative acts” at work that are collected in the questionnaire. The response form is a 5-point Likert-type scale, from 1 (never) to 5 (daily). An example of an item is item 13, “you are persistently reminded of your mistakes”. This scale in its Spanish version showed a reliability of 0.89 for the total scale, and a two-factor structure, with 0.84 for the factor of Personal Harassment and 0.81 for the one called Performance-Centered Harassment at Work. The convergent validity of this scale was elevated with other scales that measured psychosomatic problems, perceived health, absenteeism and job satisfaction. The convergent validity of this scale was elevated with other scales that measured psychosomatic problems, perceived health, absenteeism and job satisfaction.

The General Health Questionnaire (GHQ-12) of Goldberg and Williams (2000) in the Spanish adaptation of Sánchez-López and Dresch (2008) of 12 items was used to assess health. Participants must respond to the statements presented to them according to a 4-point Likert-type scale from 0 (not at all) to 3 (much more than usual). An example item is 2 “Have your worries caused you to lose a lot of sleep?”. Higher scores indicate poorer health. With this mental health assessment tool used to measure general psychological well-being and detect possible mental health problems in adults, it focuses on areas such as stress symptoms, sleep problems, ability to concentrate, mood, etc. In its Spanish version, it showed a Cronbach’s alpha of 0.76 and a 3-factor structure (with oblique rotation and the maximum likelihood method). The external validity of Factor I (successful coping) with the ISRA was very high (0.82; Factor II, 0.70; Factor III, 0.75). The GHQ-12 has reliability and validity in the Spanish population.

Job satisfaction was evaluated using the Overall Job Satisfaction Scale by Warr et al. (1979) adapted by Pérez-Bilbao and Fidalgo (1995) OJS assesses satisfaction with different aspects of the work environment composed of 15 items (for example, item 10: “recognition obtained for a job well done”), and with 7 response options, from 1, very dissatisfied, to 7, very satisfied. Cronbach’s alpha coefficient for overall satisfaction was 0.85. A two-dimensional factor structure was 0.84 for intrinsic satisfaction and 0.77 for extrinsic satisfaction. With respect to validity, it showed significant correlations with other psychosocial work factors, such as supervision, role definition and personal relationships. It should be noted that the scores of this questionnaire indicate that higher scores are indicative of better satisfaction, in contrast to the rest of the scales, where higher scores are indicators of greater discomfort in terms of more sexual or occupational harassment or greater self-perceived health discomfort.

### 2.4. Data Analysis

For the statistical analysis, we obtained means, standard deviations, skewness and kurtosis for quantitative variables, and frequencies and percentages for qualitative variables. The normality of the quantitative variables was also tested with the Shapiro–Wilks test. If the variables do not follow a normal law, then the median and interquartile range were also calculated, and Spearman’s correlation was used to evaluate the relationship between variables. The floor and ceiling effect has also been obtained using the criterion of 17% or higher as an indicator that such an effect has occurred in the study data. The significance level of the statistical tests has been α = 0.05.

For the structural analysis of the scale, a confirmatory factor analysis was performed using the full information maximum likelihood method. The criteria for considering that the resulting structure fits the original proposed model are CFI ≥ 0.95, TLI ≥ 0.95, RMSEA ≤ 0.08 and SMRS ≤ 0.08. An exploratory factor analysis was also performed on a matrix of polychoric correlations, and the estimation of factors loadings was performed using the maximun likelihood method. Factor selection was performed with parallel analysis and Promin oblique rotation was used to obtain the factor solution.

All statistical analyses were performed with SPSS (v. 22.0) and JAMOVI (v. 2.6.23).

## 3. Results

### 3.1. Descriptive Statistics of the Scales

First, the results obtained in the different measurement scales used were analyzed. To do so, first the scores of the different subscales used were formed. In the Sexual Harassment scale measured with the SEQ, it consisted of two subscales (SEQAXD: Unwanted Sexual Attention and SEQCSX: Sexual Coercion); the descriptive analyses of the scales can be seen in Table 2, which shows that it does not follow a normal distribution and with a pronounced skewness of the data, as is quite common in health scales or psychosocial phenomena evaluated in general populations. Therefore, the floor effect is very high, and the ceiling effect is very low; however, the frequency of sexual harassment by coercion is higher (M = 10.7) than that of sexual coercion (8.72), and its floor effect is 83.3% in the latter and 50.8% in the former.

The results shown asymmetrical and non-normal distributions for all scales, according to the Shapiro–Wilk test (*p* < 0.001). The SEQ dimensions show high positive skewness and high kurtosis, with pronounced floor effects, especially in SEQCAXD (83.3%). The NAQ (Workplace Harassment) also shows high positive skewness and kurtosis. In contrast, SATLA (Job Satisfaction) exhibits slight negative skewness and kurtosis close to zero, indicating more symmetrical distributions. As for the BIETR_T (Self-perceived Health) scale, it exhibits a slight positive skewness and moderate kurtosis. These data suggest that experiences of sexual harassment and negative acts are infrequent, while job satisfaction and well-being show more balanced distributions.

In summary, the data suggest that experiences of sexual harassment and bullying behaviors (SEQ and NAQ) are relatively infrequent in the sample, with highly skewed distributions. On the other hand, job satisfaction and well-being at work (SATLA and BIETR) show more symmetrical distributions close to normality.

### 3.2. Structural Validity

A confirmatory factor analysis was performed with the structure proposed by Fitzgerald et al. (1995). The fit statistics are CFI = 0.61, TLI = 0.54, RMSEA = 0.23 and RMSR = 0.13. Since the confirmatory factor analysis did not fit the 3-factor model, an exploratory factor analysis was performed, which yielded a two-factor structure for the scale in this group (Table 3). The first factor consists of items 8 and 10 to 17, and the second factor consists of items 1 to 7 and item 9. Item 12 was eliminated because it obtained a loading below 0.40. Thus, the new proposal would start from a first factor related to Sexual Harassment by Coercion (item 17: retaliated for refusing; 15: let you know that your cooperation was necessary to be treated well; 11: subtly bribed you, etc.), and a second factor that we could call Sexual Harassment by Unwanted Sexual Attention (2: tried to talk to you about sex; 3: made ordinary sexual comments; 1: told you suggestive stories, etc.). Factor I shows high factor loadings on most items suggesting that this factor represents a well-defined dimension of sexual harassment at work (items: 8, 14, 15, 16 or 17). Factor II also shows high factor loadings (items 1, 2 or 3), which also indicates the importance of its presence in the assessment of sexual harassment.

In terms of uniqueness, the items with the lowest values (e.g., item 17: 0.104, item 2: 0.153, item 15: 0.156) indicate a higher variance explained by the model, which reinforces the robustness of the two factors. In contrast, item 9 presents the highest uniqueness value (0.633), suggesting that its variance is not as well explained by the extracted factors.

Taken together, these results support the bifactorial structure of the SEQ-W scale where Factor I represents a form of violence, based on sexual coercion, and Factor II includes unwanted sexual attention actions.

### 3.3. Internal Consistency

The internal consistency, using omega and Cronbach’s alpha coefficients, of all the scales used in the study was calculated (Table 4). The 95% confidence interval is also provided in this case. In all of them and in all the scales, it shows an adequate relationship between the construct measured and each of the items that make up each scale.

### 3.4. Concurrent and Discriminant Validity

We analyzed the concurrent and discriminant validity of the scale in terms of how it correlates with the other scales used. Thus, we expect high concurrent validity with the harassment scale and with the health scale, and discriminant validity with the job satisfaction scale. Since the scales do not follow a normal distribution, Spearman’s coefficient was used. The association between all the scales was significant and in the expected direction (Table 5). The correlations were positive and highly significant between the dimensions of sexual harassment with those of workplace harassment and (poor) health, and negative with job satisfaction.

### 3.5. Empirical Validity

We analyzed the empirical validity of the SEQ scale and its two proposed dimensions, thereby supporting the possible validity of the measurement instrument with other indicators that were included in the questionnaire such as seniority in the company and in the job, absenteeism, perceived ability and job demands. A statistically significant association was found between sexual harassment by coercion and coping with work demands of physical origin, and of the general Sexual Harassment scale with Ability to cope with work (Table 6).

Finally, Table 7 shows the means and standard deviations of the SEQ scale and its two dimensions. In this case, no significant differences were found in the SEQ scale according to socio-demographic characteristics.

## 4. Discussion

The aim of this study is to validate a questionnaire on sexual harassment in Spanish developed by Fitzgerald et al. (1995) called SEQ-W, using a sample of Spanish-speaking female workers. The validation of a new version of the SEQ-W in Spanish for the workplace is important due to the need for cultural and linguistic adaptation with adequate psychometric properties that guarantee its reliability and validity for use in organizational contexts where there is a legal framework, both preventive and punitive, that prevents the occurrence of these episodes of harassment, which, as we have mentioned, have a high impact on the health of those who suffer them.

This questionnaire was chosen because it has served as an instrument to assess prevalence in organizations or as forensic evidence in cases of legal complaints filed by women victims of sexual violence (Gutek et al., 2004).

Confirmatory factor analysis has not supported the original three-dimensional structure in this group. The failure of CFA to replicate the original structure can be explained, first, by the insufficient sample size, which reduces the stability of the estimates and the ability to replicate complex structures. In addition, cultural differences may modify the interpretation of items and the emergence of factors, as has been observed in previous validations in Mexico and South America, where the factor structure differs from the original. For example, Fitzgerald et al. (1995) reported difficulties with the sexual coercion dimension (low internal consistency), and other validations have found bifactorial structures or different groupings of items. It is proposed that future research include qualitative analyses to explore the understanding of the items and further investigate the influence of sociocultural factors.

However, an exploratory factor analysis has suggested a two-dimensional structure, representing the most common forms of workplace harassment: sexual harassment by coercion and harassment by unwanted sexual attention. Both dimensions showed adequate internal consistency, concurrent validity with workplace harassment scales and psychological well-being, and discriminant validity with job satisfaction. This factor solution raises questions about the universality of the three original dimensions of the SEQ-W authors and supports the need for adequate validations in different linguistic contexts.

In the version developed by Fitzgerald et al. (1995) designed for workers and derived from the previous 1988 version, 17 items are collected and factorized into three dimensions with adequate psychometric properties through confirmatory analysis. The complete and pre-SEQ-W versions (with 18 and 26 items) showed internal consistency between 0.70 and 0.90. However, in the three proposed dimensions, the internal consistency coefficients were 0.82 for gender-based harassment, 0.85 for unwanted sexual attention, and 0.42 for sexual coercion (Gutek et al., 2004). It is possible that sexual coercion is a much less frequent type of sexual harassment and that there is little variability in the responses, which reduces the covariance between the items. This makes a two-factor structure possible, as presented in our article. In addition, item 12 was removed due to a factor loading of less than 0.40, indicating a low relationship with the identified dimensions. In theory, this item may be less aligned with the predominant forms of harassment in the Spanish-speaking context, or its wording may lead to interpretive ambiguity. We therefore recommend revising this item and possibly reformulating it in future applications, as well as conducting qualitative analyses (clinical interviews) to explore participants’ understanding of it. Item 9 showed high uniqueness, suggesting that its variance is not well explained by the extracted factors. We also recommend reformulating it to improve its alignment with the corresponding dimension or conducting a qualitative analysis to determine whether it reflects a relevant but infrequent or poorly understood experience.

Regarding validity measures, the data support adequate fit with content, construct, and criterion validity (Gutek et al., 2004).

In summary, the different versions developed with the SEQ already show the need to adapt to different cultural settings, and their factorial structure does not match the one initially proposed.

### 4.1. Practical Implications

The results suggest that the SEQ-W scale can be used in samples of Spanish-speaking workers with a two-dimensional structure: Coercive Sexual Harassment and Unwanted Sexual Attention Harassment.

With this scale, a screening questionnaire on sexual harassment can be used in a clinical or organizational context to identify people who have been victims of sexual harassment to intervene in its possible psychological consequences (depression, PTSD, or anxiety).

On the other hand, helping professionals to assess the magnitude and severity of the behaviors to which the victim feels harassed can be of great value from several perspectives. From the list of behaviors, it can facilitate the emotional expression of the experienced experiences.

In organizational contexts, it can help to identify patterns of harassment and improve the preventive culture of zero tolerance with this type of bullying attitudes and behaviors, and that sometimes, we are not aware of how permissive we are with some of “low intensity”.

And finally, it can facilitate a differential diagnosis, both with other forms of harassment (workplace harassment, harassment based on ethnicity, race or any other condition) and in the categories of harassment by coercion and unwanted sexual attention.

### 4.2. Limitations

The study has several limitations that should be taken into account when interpreting the results. First, the sample consisted of 120 female workers in the service, education, healthcare, and public administration sectors in Spain and South America (Ecuador and Colombia). The geographical distribution may influence the results due to regulatory, cultural, and organizational differences. The use of a convenience sample and the absence of male participants limit the generalizability of the results. It is therefore recommended that future research include men and diversify the occupational sectors to improve the representativeness of the sample.

The sample size (N = 120) is a significant limitation, especially for confirmatory factor analysis (CFA). A small sample size can affect the stability of factor solutions and the ability to detect complex latent structures, as well as reducing statistical power and increasing the likelihood of type II errors. This may have contributed to the failure of CFA to replicate the original three-factor structure. Although increasing the sample size is not feasible at this stage, future studies are recommended to include larger and more diverse samples, which would allow for multigroup analysis and greater robustness in the cross-cultural validation of the instrument.

The use of a non-probability convenience sampling method may introduce selection bias. Although an effort was made to include participants from diverse organizational settings, information on specific types of companies or sectors was not systematically collected, limiting the ability to assess contextual influences such as organizational culture. Furthermore, the cross-sectional design of the study prevents causal inferences from being drawn between the variables analyzed. Finally, data were obtained through self-report measures, which may be influenced by social desirability and recall bias.

Future research should aim to include larger and more diverse samples, incorporate male participants to assess the broader applicability of the scale, and consider longitudinal designs and multiple sources of data to improve validity and generalizability.

### 4.3. Future Lines of Research

Validation studies: Conduct additional validation studies with larger and more diverse samples of Spanish-speaking workers, including men and people of different ages, educational levels and occupations.

Longitudinal studies: Conduct longitudinal studies to examine the relationship between sexual harassment and other relevant variables over time, such as psychological well-being, job satisfaction and job performance.

Qualitative analysis: Combine quantitative and qualitative methods to gain a deeper understanding of sexual harassment experiences in the Spanish-speaking workplace, including interviews and focus groups.

Cross-cultural comparison: Conduct comparative studies across cultures and countries to examine similarities and differences in the structure and prevalence of sexual harassment, as well as its consequences and associated factors.

Interventions: Develop and evaluate the effectiveness of interventions designed to prevent and address sexual harassment in the workplace, using an evidence-based approach tailored to the characteristics of the target population.

Risk factors: To deepen research on individual, organizational and sociocultural factors that contribute to sexual harassment in the workplace, to design more effective prevention strategies.

## Figures and Tables

**Table 1:** Socio-demographic characteristics of the group.

	f (%)	M (DT)	R
Age		33.9 (10.5)	[21–75]
Genre	120 (100)		
Nationality			
European	81 (67.5)		
South America	39 (32.5)		
Marital status			
Married	40 (33.6)		
With partner	21 (17.6)		
Single	52 (43.7)		
Others	6 (5.0)		
Studies			
Unqualified	2 (1.7)		
Primary	4 (3.3)		
Secondary	27 (22.5)		
University students	87 (72.5)		
Type of contract			
Indefinite	70 (58.3)		
Temporary	45 (37.5)		
Others	5 (4.2)		
Type of shift			
Morning or afternoon	33 (27.5)		
Morning and afternoon	67 (55.8)		
Others	20 (16.7)		
Type of company			
Public	30 (25)		
Private	90 (75)		
Position in the company			
Management	18 (15)		
Intermediate control	23 (19.2)		
Technician	79 (65.8)		
Seniority in the company		6.01 (6.42)	[0–30]
Seniority in the position		4.01 (4.37)	[0–21]
Days of sick leave		3.01 (12.6)	[0–120]
Capacity		8.89 (1.42)	[1–10]
Physical Demand		4.33 (0.65)	[1–5]
Psychological Demand		4.13 (0.76)	[1–5]

Notes: f = frequency, % = percentage, M = mean, SD = standard deviation, R = range.

**Table 2:** Descriptive statistics of the scales in this study.

	l	M (DT)	Md (RIC)	Min	Max	Bias	Kurtosis	SH-W	Effect Floor	Effect Ceiling
SEQCAXD	8	8.72 (2.53)	8 (0.0)	8	25	4.84	25.0	0.318 ***	83.3%	0.8%
SEQASXD	8	10.7 (4.56)	8 (3.0)	8	33	2.42	6.50	0.649 ***	50.8%	0.8%
SEQ_T	16	19.4 (6.40)	16.5 (4.0)	16	53	2.83	8.95	0.594 ***	50%	0.8%
NAQ.APD	13	16.4 (4.17)	15 (5.0)	13	38	2.44	8.94	0.759 ***	----	----
NAQ.ATD	10	16.3 (6.55)	15 (8.0)	10	39	1.56	2.57	0.838 ***	----	----
NAQ_T	23	32.8 (10.0)	30 (12.0)	23	77	1.83	4.92	0.836 ***	----	----
SATLIND	7	36.0 (9.51)	37 (13.0)	11	49	−0.794	0.097	0.934 ***	----	----
SATLEXD	8	41.2 (8.73)	42 (11.0)	20	56	−0.446	−0.314	0.973 ***	----	----
SATLA_T	15	77.2 (17.6)	78 (22.0)	35	105	−0.605	−0.113	0.957 **	----	----
BIETR_T	12	21.8 (6.22)	6.22 (8.0)	12	42	0.83	0.80	0.95 ***	----	----

Notes: SEQASEXD = Dimension 1 of the SEQ scale, SEQCSXD = Dimension 2 of the SEQ scale, SEQ_T = Total score on the SEQ scale, NAQAPD = Dimension 1 of the NAQ scale, NAQATD = Dimension 2 of the NAQ scale, NAQ_T = Total score on the NAQ scale, SATLIND = Dimension 1 of the SATLA scale, SATLEXD = Dimension 2 of the SATLA scale, SATLA_T = Total score on the SATLA scale, BIETR_T = Total score on the BIETR scale, l = number of items of the dimension/scale, M = Mean, SD = Standard deviation, Md = Median, IQR = Interquartile range, Min = Minimum, Max = Maximum, SH-W = Shapiro–Wilk test, ** *p* < 0.01, *** *p* < 0.001.

**Table 3:** Factor structure of the SEQ scale.

	Factor I	Factor II	Uniqueness
Item 1		0.821	0.278
Item 2		0.957	0.153
Item 3		0.913	0.249
Item 4		0.582	0.571
Item 5		0.661	0.258
Item 6		0.557	0.536
Item 7		0.485	0.525
Item 8	0.809		0.273
Item 9		0.591	0.633
Item 10	0.618		0.295
Item 11	0.828		0.386
Item 13	0.506		0.516
Item 14	0.722		0.421
Item 15	0.926		0.156
Item 16	0.763		0.330
Item 17	0.990		0.104

**Table 4:** Internal consistency coefficients (α and ω) of the scales.

	α	IC	ω	IC
SEQCAXD	0.91	[0.88–0.93]	0.92	[0.77–0.97]
SEQASXD	0.90	[0.87–0.92]	0.91	[0.85–0.94]
SEQ_T	0.92	[0.89–0.93]	-----	------
NAQAPD	0.80	[0.75–0.84]	0.83	[0.65–0.90]
NAQATD	0.89	[0.86–0.92]	0.90	[0.84–0.93]
NAQ_T	0.91	[0.89–0.93]	-----	------
SATLAIND	0.92	[0.90–0.94]	0.92	[0.87–0.95]
SATLAEXD	0.86	[0.82–0.89]	0.87	[0.80–0.91]
SATLA_T	0.94	[0.92–0.95]	-----	------
BIETR_T	0.89	[0.86–0.92]	0.90	[0.86–0.93]

Notes: SEQAXD = Sexual Coercion Harassment from the SEQ scale, SEQASXD = Sexual Attention Unwanted from the SEQ scale, SEQ_T = Total score on the SEQ scale, NAQAPD = Personal Workplace Harassment from the NAQ scale, NAQATD = Job Performance Workplace Harassment 2 from the NAQ scale, NAQ_T = Total score on the NAQ scale, SATLIND = SATLA scale Intrinsic Satisfaction 1, SATLAEXD = SATLA scale Extrinsic Satisfaction, SATLA_T = SATLA scale total score, BIETR_T = BIETR scale total score, l = number of items in the dimension/total scale, α = Cronbach’s alpha coefficient, ω = McDonald’s omega coefficient, CI = 95% confidence interval.

**Table 5:** Spearman correlations between the NAQ, SAT-LA, BIETR and SEQ scales.

	(1)	(2)	(3)	(4)	(5)	(6)	(7)	(8)	(9)
(2)	0.77 ***								
(3)	0.90 ***	0.97 ***							
(4)	−0.64 ***	−0.77 ***	−0.76 ***						
(5)	−0.59 ***	−0.72 ***	−0.71 ***	0.87 ***					
(6)	−0.63 ***	−0.77 ***	−0.76 ***	0.96 ***	0.97 ***				
(7)	0.49 ***	0.56 ***	0.56 ***	−0.62 ***	−0.53 ***	−0.59 ***			
(8)	0.35 **	0.25 *	0.28 *	−0.25 *	−0.30 ***	−0.27 *	0.19 *		
(9)	0.52 ***	0.45 ***	0.50 ***	−0.42 ***	−0.36 ***	−0.40 ***	0.28 **	0.58 ***	
(10)	0.51 ***	0.44 ***	0.49 ***	−0.41 ***	−0.37 ***	−0.40 ***	0.27 **	0.63 ***	0.99 ***

Notes: (1) NAQAPD = NAQ Personal Work Harassment, (2) NAQATD= NAQ Job Performance Harassment at Work, (3) NAQ_T = Total score on the NAQ scale, (4) SATLAIN= SATLA Intrinsic Satisfaction, (5) SATLEX= SATLA Extrinsic Satisfaction, (6) SATLA_T = Total score on SATLA scale, (7) BIETR_T = Total score on BIETR scale, (8) SEQCSXD = SEQ Coercive Sexual Harassment, (9) SEQASXD = SEQ Unwanted Sexual Harassment scale (10) SEQ, SEQ_T = Total score on SEQ scale, * *p* < 0.05, ** *p* < 0.01, *** *p* < 0.001.

**Table 6:** Spearman correlations of SEQ subscales and other study variables.

	(1)	(2)	(3)	(4)	(5)	(6)
(2)	0.77 ***					
(3)	0.09	−0.00				
(4)	−0.10	0.07	−0.09			
(5)	0.03	0.10	−0.03	0.53 ***		
(6)	−0.08	−0.04	0.03	0.58 ***	0.63 ***	
(7)	−0.01	−0.01	0.12	−0.14	−0.21 *	−0.05
(8)	−0.07	−0.08	0.11	−0.18	−0.12	−0.12
(9)	−0.05	−0.07	0.10	−0.19*	−0.15	−0.11

Notes: (1) Length of service, (2) Length of service, (3) Days of sick leave, (4) Ability, (5) Physical demand, (6) Psychological demand, (7) SEQCSX1 subdimension, (8) SEQASX subdimension, (9) SEQ total score, * *p* < 0.05, *** *p* < 0.001.

**Table 7:** Means and standard deviations (medians and interquartile range) of the subscales of the SEQ scale as a function of group characteristics.

	SEQCSXD		SEASXD		SEQ_T	
	M (DT)	Md (RIC)	M (DT)	Md (RIC)	M (DT)	Md (RIC)
Nationality						
European	8.7 (2.6)	8.0 (0.0)	10.9 (4.7)	9.0 (3.0)	19.5 (6.6)	17.0 (4.0)
South America	8.9 (2.5)	8.0 (0.0)	10.3 (4.3)	8.0 (3.0)	19.2 (6.1)	16.0 (3.5)
Marital status						
Married	8.5 (1.9)	8.0 (0.0)	9.45 (2.4)	8.0 (2.0)	17.9 (4.1)	16.0 (2.0)
With partner	8.5 (1.1)	8.0 (0.0)	11.4 (6.6)	9.0 (2.0)	19.9 (7.6)	17.0 (4.0)
Single	8.9 (3.2)	8.0 (0.0)	11.4 (4.9)	9.0 (4.3)	20.3 (7.2)	17.0 (6.0)
Others	9.7 (4.1)	8.0 (0.0)	10.5 (3.5)	9.5 (3.0)	20.2 (7.4)	17.5 (3.0)
Studies						
Unqualified	8.0 (0.0)	8.0 (0.0)	8.0 (0.0)	8.0 (0.0)	16.0 (0.0)	16.0 (0.0)
Primary	8.0 (0.0)	8.0 (0.0)	11.0 (3.5)	10.0 (2.0)	19.0 (3.5)	18.0 (2.0)
Secondary	8.9 (3.4)	8.0 (0.0)	11.3 (6.2)	9.0 (3.5)	20.3 (8.9)	17.0 (3.5)
University students	8.7 (2.3)	8.0 (0.0)	10.5 (4.1)	8.0 (3.0)	19.2 (5.6)	16.0 (4.0)
Type of contract						
Indefinite	8.4 (1.4)	8.0 (0.0)	10.4 (4.0)	8.0 (3.0)	18.9 (4.9)	16.5 (3.8)
Temporary	9.2 (3.7)	8.0 (0.0)	11.2 (5.5)	9.0 (4.0)	20.5 (8.4)	17.0 (4.0)
Others	8.0 (0.0)	8.0 (0.0)	9.0 (2.2)	8.0 (0.0)	17.0 (2.2)	16.0 (0.0)
Type of shift						
Morning or afternoon	8.8 (2.1)	8.0 (0.0)	10.8 (5.5)	8.0 (3.0)	19.5 (7.0)	16.0 (4.0)
Morning and afternoon	8.9 (3.0)	8.0 (0.0)	11.1 (4.6)	9.0 (4.0)	19.9 (6.9)	17.0 (4.0)
Others	8.2 (0.5)	8.0 (0.0)	9.3 (1.5)	9.0 (2.0)	17.4 (1.9)	17.0 (2.0)
Type of company						
Public	9.0 (2.7)	8.0 (0.0)	11.1 (4.4)	9.0 (4.0)	20.1 (6.2)	17.0 (4.0)
Private	8.6 (2.5)	8.0 (0.0)	10.5 (4.6)	8.0 (3.0)	19.2 (6.5)	16.0 (3.0)
Position in the company						
Address:	8.1 (0.2)	8.0 (0.0)	9.0 (1.7)	8.0 (1.0)	17.1 (1.8)	16.0 (1.0)
Intermediate control	8.9 (3.1)	8.0 (0.0)	10.5 (4.2)	8.0 (3.0)	19.4 (6.7)	16.0 (3.0)
Technician	8.6 (1.1)	8.0 (0.0)	12.6 (6.3)	10.0 (9.0)	21.1 (7.3)	18.0 (9.0)

Notes: SEQCSXD = Total score Coerced Sexual Harassment from the SEQ scale, SEQASXD = Total score Unwanted Sexual Harassment from the SEQ scale, SEQ_T = Total score on the SEQ scale.

## Data Availability

Data supporting the findings of this study are available upon request from the corresponding author.
